# Transforming GP training in the UK: the lasting impact of COVID-19 on telehealth and hybrid care models

**DOI:** 10.3389/fmed.2025.1595937

**Published:** 2025-06-04

**Authors:** Waseem Jerjes, Azeem Majeed

**Affiliations:** Department of Primary Care and Public Health, Faculty of Medicine, Imperial College London, London, United Kingdom

**Keywords:** GP training, telehealth, hybrid care models, artificial intelligence in primary care, digital literacy, medical education reform

## Introduction

The COVID-19 pandemic not only marked a public health crisis but a turning point in the practice of medicine and medical education (1). While many sectors, including education, training, and business meetings, also moved rapidly online, no location felt this shift as starkly as general practice, where traditional in-person consultations were rapidly replaced by remote consultations (2, 3). The shift came out of necessity, but as emergency measures were formalized into standard practice, it became clear that telehealth and blended care models would be central to future primary care (1, 4). This overnight restructuring, as much as it was unavoidable, brought to light critical gaps in GP training, leaving trainee doctors to master new technologies and patterns of consultation without any structured framework to do so.

The second step is to evolve out of reactive adaptation and develop a strategic, future-proof GP training system that integrates digital health, artificial intelligence (AI), and hybrid care in full, yet maintains the essence of patient-centered medicine (2, 3). The outdated apprenticeship model of GP training based on observation and direct contact with patients is insufficient when virtual consultations, AI-assisted triage, and remote monitoring are becoming the new reality. GP trainees should not only be skilled in person-to-person medicine, but also on telehealth platforms, digital diagnosis, and ethical decision-making in an AI environment.

This view point addresses how GP training must evolve to meet the needs of a hybrid healthcare system. It introduces the Hybrid GP Training Model, a structured method of blending digital and in-person learning so that trainees acquire skills to function proficiently in both settings. It will also address how AI is going to supplement GP training, the significance of interprofessional working, the global implications of telehealth training, and the ethical dilemmas of a digital-first healthcare system. It will then provide policy and education reform suggestions, suggesting a national telehealth competency framework and systematic digital literacy training for GP trainees.

As general practice is at a turning point, the question is not so much whether telehealth and AI should be included in training as how to include it without losing the very essence of general practice. The question is how to build a new generation of GPs who are not only technologically proficient but who have undergone structured GP training to remain clinically competent, adaptable, and highly attuned to the humanity of medicine.

## Beyond crisis management: the hybrid GP model of training

The emergency adoption of telehealth during the COVID-19 pandemic is over, yet its future role in daily practice needs a more structured, forward-thinking training solution (4–6). The challenge is now to ensure that GP trainees not only learn through experience, but learn systematically to deal with remote and face-to-face consultations with equal proficiency. We require a Hybrid GP Training Model that establishes telehealth as a core skill without forgetting that practical clinical skills should be central to medical training.

The shift to digital-first care changed the manner in which GP trainees develop key skills. Table 1 summarizes the core components of the Hybrid GP Training Model, highlighting the key training areas, the competencies required, and their purpose within a hybrid practice environment. In the past, training centered on face-to-face consultations, where judgment skills were developed through direct communication with patients, physical examination, and on-the-fly guidance (7, 8). With virtual consultations now becoming the new reality, however, trainees were practicing in an environment where diagnostic clues were limited to a voice, facial expressions on a screen, or text in an e-consult. While these skills have since become essential, they have been acquired informally rather than through structured training, with inconsistent competency being the result.

**Table 1:** Core components of the hybrid GP training model.

**Training area**	**Core skills developed**	**Purpose in hybrid practice**
Digital communication	Conducting video, telephone, and e-consultations	Ensure effective and empathetic remote interactions
Remote clinical reasoning	Diagnosis and management without physical examination	Maintain diagnostic safety and confidence in virtual care
In-person clinical skills	Bedside examination, clinical judgment, rapport-building	Preserve face-to-face consultation excellence
Digital literacy and AI proficiency	Navigating telehealth systems, interpreting AI tools and outputs	Safely integrate technology into clinical workflows
Ethical decision-making	Recognizing AI bias, protecting patient data, informed consent	Uphold ethics and safeguard patient trust in digital care
Health equity awareness	Identifying and addressing digital exclusion and disparities	Ensure equitable access to care across patient populations

**Figure 1:**
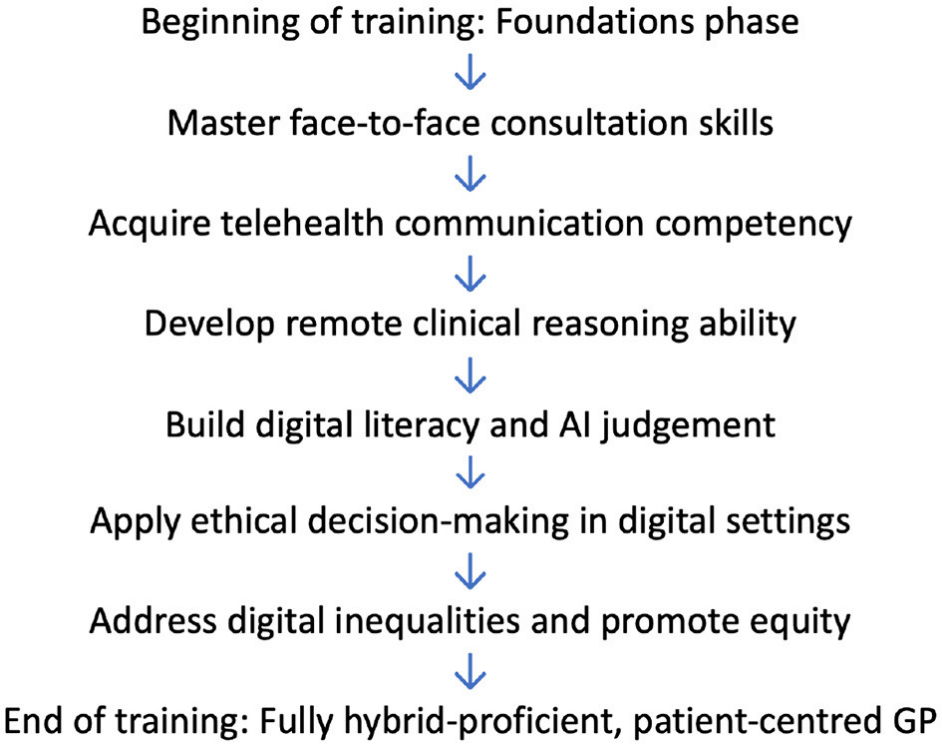
Developmental stages in the hybrid GP training model. This figure illustrates the staged progression of GP trainee development within a hybrid training framework. It begins with foundational in-person consultation skills and gradually incorporates telehealth communication, remote clinical reasoning, digital literacy, ethical practice in digital settings, and attention to health equity. The outcome is a fully hybrid-proficient, patient-centered GP.

A Hybrid GP Training Model would establish specific learning outcomes and assessment criteria for telehealth so that trainees acquire the ability to provide remote consultations without losing the ability to transition to in-person care (9, 10). Figure 1 illustrates the staged development of core competencies throughout the Hybrid GP Training Model, from foundational consultation skills to advanced digital equity and AI judgement. The model needs to include specific training in digital communication, remote environment decision-making, and systematic training on when an in-person examination is needed. Additionally, a Digital Readiness Index for GP trainees needs to be applied, evaluating the trainee's skill in telehealth tools, AI-assisted decision-making, and remote patient interaction.

Where telehealth has brought increased access, it has also introduced new challenges, not least in patient relations (7). Physical absence is likely to lead to a more transactional style of interaction, where issues may be addressed economically but less sensitively. GP trainees should be trained to adapt consultation style to the remote context so that patient-centered care is not compromised in the interests of efficiency. A systematic hybrid approach to training would deal with the skills of working with the nuances of remote cues, the use of strategic questioning to compensate for the lack of physical examination, and building rapport in a remote context.

Besides clinical ability, hybrid training should also address inequalities in healthcare caused by digital healthcare (6). Not all individuals are on the same level when it comes to access to technology, and some do not have digital skills, so remote consultations end up being a barrier rather than an enabler of care. GP trainees should be taught to be aware of these issues and use an equity approach to telehealth so that online consultations do not widen inequalities in accessing healthcare.

Success with telehealth in the long-term within the context of primary care depends not on ongoing adjustment, but on active incorporation into GP training (10). By incorporating telehealth skills into curricula, developing structured blended learning opportunities, and assessing trainees on both digital and face-to-face care, the Hybrid GP Training Model provides a template for training GPs to meet a future in which technology enhances rather than replaces high-quality care.

## Telehealth training and AI in medical education

The integration of telehealth into GP training should be underpinned by systematic, evidence-based methods so that online consultations meet the same standard as consultations in person (11, 12). However, traditional medical education has not kept pace with technological advances, so telehealth skills have been acquired by many GP trainees through experience and error. To fill this gap, artificial intelligence (AI) and digital simulation hold promising solutions, complementing telehealth training with immediate feedback, systematic assessment, and immersive environments.

Among the most promising of the innovations is consultation training supported by AI, where AI tools review a trainee's actual consultation in real time, assessing communication skills, diagnostic accuracy, and decision-making. With immediate feedback on language, tone, and interaction with patients, AI tools can refine a trainee's ability to build rapport remotely as well as diagnostic accuracy (2). AI-supported platforms also hold the potential to simulate actual patient encounters so that trainees can practice remote history-taking and diagnostic thinking within a simulated environment prior to seeing actual patients.

Besides AI-aided training, augmented reality and digital twins are the key to bridging the remote and on-site assessment (13, 14). With augmented reality, remote observation and supervision of GP trainees is possible, with instant guidance in digital consultations. Meanwhile, digital twin technology, where a virtual representation of a patient is created based on real-time data, is likely to enable trainees to develop clinical reasoning on complex cases before actually seeing the patients. Such technologies could transform GP training so that it is not just convenient, but clinically effective.

Despite these advantages, AI-aided training also presents ethical and practical issues that should be considered (14). One of the potential pitfalls is over-reliance on AI decision-support systems, where trainee GPs depend on machine suggestions without critically assessing independent clinical judgment. This could compromise diagnostic confidence and hinder uncertainty handling in the clinic—a key set of skills demanded of GPs. Additionally, AI models are as biased as the data on which they have been trained; if digital training tools are built on data that do not represent diverse groups of patients, then they risk reinforcing instead of alleviating disparities in care.

For integration to be effective, AI should be used as a complement to education rather than a substitute for experience (12). AI literacy needs to be woven into training programs so that trainees learn to critically assess AI-driven findings and know its limitations. AI should not be viewed as a substitute for traditional learning, but rather as an additional tool that enhances decision-making and refines clinical judgment without losing the humanness of medicine.

The telehealth training should be infused with the technological advances in a manner that is grounded in the core values of person-centered care. With the incorporation of AI-aided consultation feedback, augmented reality-enhanced learning, and digital simulations of patients, GP training can be revised so that remote consultations are as clinically sound and compassionate as face-to-face encounters.

## Interdisciplinary collaboration: the future of GP training

The future of general practice is not merely about technical expertise; it is about working across disciplines to equip GP trainees with a broad set of skills relevant to modern primary care. The shift toward hybrid care models, AI-enabled consultations, and telehealth-delivered mental health care means GP trainees cannot be trained in isolation (15). For holistic competence, GP training needs to include learning alongside public health professionals, digital health specialists, and clinical psychologists.

One of the areas where cooperation across disciplines is critical is mental health training in telehealth consultations (16–18). The pandemic has led to increased anxiety, depression, and post-traumatic stress disorder, so mental health is a growing part of daily general practice. Remote consultations, however, present certain challenges in the detection of distress, assessment of suicide risk, and effective psychological intervention. In collaboration with clinical psychologists, GP trainees should be formally trained in online mental health assessment, learning skills in detecting nuances of distress and using structured interviewing skills to overcome the lack of direct interaction. If not, telehealth is likely to be an inadequate substitute for mental health care in person, leaving vulnerable patients under-served.

Besides mental wellbeing, it is essential to partner with digital health professionals so that GP trainees acquire the skills necessary in the rapidly expanding field of AI-aided diagnosis, wearable technologies, and digital literacies (16–18). With AI tools increasingly being applied to assist diagnosis and remote monitoring, GP trainees must learn how to integrate these technologies into practice without being too reliant on algorithmic recommendations. Digital health professionals will help trainees acquire key AI literacies so that they learn to interpret AI-derived insights without compromising on patient safety, ethics, and clinical judgment.

Public health training is also a critical component of a future-proof GP training programme. The COVID-19 pandemic brought the role of primary care in crisis response into sharp focus, yet very few GP trainees were formally trained in pandemic preparedness, managing misinformation on health issues, and promoting community health resilience (18, 19). The modern GP training programme should include dual learning with public health professionals so that the trainees acquire the skills to handle future crises, be it infectious diseases, climate-induced health threats, or mass casualty events. Coordinating public health interventions, handling vaccine hesitancy, and implementing rapid response measures should be a core component of GP training.

Interprofessional working not only enhances clinical ability, but also changes the identity of the modern GP into digital navigators of health, mental first responders, and public health advocates on the frontline (17, 19). Breaking down the traditional silos and combining co-training with psychologists, digital health professionals, and public health specialists allows GP education to break free of its conventional frames. It will not only make future GPs excellent clinicians, but also adaptable leaders in the complex healthcare landscape.

## The global dimension: positioning UK GP training as model for the future

The UK's redesign of GP training provides the opportunity to set a global standard for digital-first, blended medical education. While telehealth was hard to roll out across much of the healthcare system during the COVID-19 pandemic, the UK has since made remote consultations an integral part of standard general practice (20, 21). This is not unique to the UK, however—the telehealth and blended care phenomenon has now gone worldwide, with countries at varying stages of adoption. With further codification and development of the Hybrid GP Training Model, the UK has the potential to set the standard for an exportable, scalable solution that enhances primary care training worldwide.

One potential route to global leadership is telemedicine training across borders, whereby GP trainees undergo overseas virtual mentorships and knowledge exchanges (22). The potential to remotely consult across borders might introduce UK trainees to global patterns of illness, varied healthcare infrastructures, and different models of providing first-contact care. For example, a UK GP trainee teleconsulting with a low-resource setting patient via telemedicine would develop skills in diagnostic thinking without high-tech investigations, cross-cultural communication, and remote prescribing on limited formularies. Such experience not only enhances clinical skills but also advances global health equity by opening up access to GP-led telehealth consultations in remote locations.

Besides telemedicine, the UK's digital approach to GP training is something that other medical education programmes around the world could learn from Ayers et al. (23) and Hindelang et al. (24). Countries with incipient telehealth infrastructures might learn from a structured GP training syllabus comprising:

- AI-aided consultation training to improve remote diagnostic accuracy
- Digital skills training to enable competency on telehealth platforms
- Simulation learning to develop remote consultation skills within a controlled environment,
- Crisis response training to provide skills to GPs in public health emergencies.

Such a framework could be implemented and applied across different healthcare systems so that GPs across the globe are trained not just as clinicians, but as leaders of digital and hybrid care.

However, with global expansion comes the burden of digital equity. While the UK is ahead of the curve when it comes to telehealth uptake, the rest of the globe is held back by disparities in accessing digital healthcare ranging from inadequate internet connectivity to low health literacy and economic constraints (21). GP trainees should be trained to be sensitive to these disparities and come up with strategies of inclusive telehealth delivery. Future GPs working in the NHS or internationally should make it a point to ensure blended care models do not exacerbate inequalities but rather facilitate bridging healthcare gaps.

By setting the UK's developing GP training model as a global standard, it is not only possible to bring medical education in the country up to date, but also to help set the future of primary care worldwide. Future generations of GPs should be trained to practice in an increasingly global healthcare environment where digital consultations, working internationally, and AI-driven care are not the exception, but the norm.

## Ethical concerns in digital-first GP training

As telehealth and AI-aided decision-making come to the fore in GP training, the ethical landscape of primary care is being rewritten (25). While the enhancements bring unprecedented efficiency, accessibility, and diagnostic aid, they also introduce profound ethical issues that have to be addressed in order not to produce unintended consequences. The Hybrid GP Training Model will require integrating ethical consideration as a core competency so that future GPs possess the skills to reconcile the role of technology with person-centered care.

One of the largest concerns is depersonalization risk in telehealth consultations. Telehealth has improved access to care, especially in remote and mobility-constrained patients, but it is increasingly worried that it could weaken the depth of the doctor-patient connection (26, 27). It is simpler to overlook the facial expressions, posture, and other non-verbal cues of unease, uncertainty, or emotional distress in a remote setting. GP trainees therefore have to learn how to proactively make up for the loss of in-person dynamics using advanced questioning skills and digital communication techniques to maintain patient trust and engagement.

AI-supported decision-making presents yet another ethical issue. While AI systems have the potential to aid diagnosis, alert clinicians to potential clinical risk, and streamline administration, over-reliance on algorithmic advice is a risk. GP trainees must not merely learn how to utilize AI, but when to challenge it, so that clinical judgment is always paramount in care (26, 28). Algorithmic bias is yet another issue—if AI systems learn on data that is not representative of the range of patients seen in practice, then they have the potential to perpetuate inequalities in health rather than mitigate them. If GPs have poor AI literacy, then they will perpetuate biased decision-making without even knowing it, disproportionately affecting vulnerable groups.

Disparities in digital healthcare extend beyond AI. The rushed shift to telehealth has already widened disparities in access, as individuals lacking digital skills, stable internet connection, or owning smart devices face significant barriers to remote care (27, 29). GP trainees need to be taught to be sensitive to and mitigate these disparities so that the expansion of digital healthcare does not leave vulnerable groups behind. This could involve creating hybrid consultation pathways, where digital care is made accessible but not at the cost of traditional, in-person access by those who need it.

Both confidentiality and data security are also essential concerns in a digital-first training environment (27). With remote consultations becoming more common amongst GP trainees, data storage, security, and sharing is becoming increasingly important. Cybersecurity principles, ethical data handling, and consent in telehealth should be taught to trainees so that technological advancement is not at the expense of privacy and trust.

Last, the Hybrid GP Model should not just be about technological competence, but also about the ethics of digital care (29). Future GPs should be trained to critically evaluate the limitations of AI, the risk of digital exclusion, and the responsibility to maintain a patient-centered approach amidst a rapidly transforming healthcare environment. The integration of bioethics, digital literacy, and health equity training into GP training will be critical in preparing clinicians to navigate not just the technical, but also the ethical challenges of modern-day primary care.

## Policy and educational reform: the next steps

Reforming GP training in preparation for telehealth and blended care requires proactive action at both the level of education and policy (30, 31). Although the COVID-19 pandemic accelerated the roll-out of remote consultations, GP training programmes have been reactive rather than proactive in terms of change. If the intention is to build a digitally literate, ethically aware, and adaptable primary care workforce, then coordinated, long-term change is required at both institutional and national levels.

A critical first step is the creation of a national telehealth competency framework, with defined, standardized learning outcomes in digital consultation skills, AI-aided decision-making, and blended care delivery (6, 30, 32). GP trainees learn telehealth skills currently on an informal experience-only basis rather than through formal training. This is not sufficient in a healthcare system where digital consultations are not just an emergency solution but a part of the future. A competency framework will instill baseline competence in telemedicine etiquette, remote diagnostic reasoning, and virtual physical assessment skills prior to trainee qualification.

There is also a necessity to invest in simulation telehealth training. Traditionally, medical training has relied on in-person case-based learning, but a blended care model requires new digital learning environments (30). Virtual patient simulations, AI-facilitated consultation feedback mechanisms, and augmented reality remote supervisions should be implemented in GP training curricula so that trainee doctors gain structured telehealth experience before consulting patients remotely. Medical schools and GP training schemes should be funded to develop and roll out these digital learning tools at scale.

No less important is the integration of digital literacy into GP training. It is not merely a question of telehealth system operation learning—trainees must be taught critical thinking skills to assess AI-driven clinical recommendations, comprehend digital diagnostic tools, and navigate ethical dilemmas in online care (31). This should include dedicated education on AI bias, healthcare cybersecurity, and patient-centered digital care models. If GPs are to be leaders in integrating digital health, then their training needs to be attuned to the nuances of this role.

Another key reform is mandatory training in hybrid consultations. Whereas, telehealth is ubiquitous, there is still no universal requirement on GP trainees to be assessed on remote consultations before being qualified (30, 32). Formal telehealth rotation—in which trainees have allocated time to perform supervised online consultations with genuine patients—should be a standard part of GP training so that digital skills are learned in a systematic, reflective context and not on the job.

Policy makers, professional associations, healthcare regulators, and medical educators will have to collaborate to introduce these changes. NHS England, Royal College of General Practitioners (RCGP), and General Medical Council (GMC) will have to join forces to establish telehealth training standards, provide funding to simulation training, and include digital skills in medical education. If not, the accelerated digitalisation of primary care is bound to leave behind professionals who will be required to provide it, with future GPs not having the skills to deal with the reality of hybrid care.

The future of GP training needs to include digital innovation without forgetting the core values of patient-centered care. With systematic reform—from competency frameworks to telehealth training through simulation and AI literacy education—the UK is poised to set a gold standard of modern GP training so that primary care is not just technologically advanced, but clinically strong, ethically outstanding, and accessible to all patients.

## Discussion: the future of GP training in an AI-driven, hybrid healthcare system

The rapidly accelerated change in general practice, prompted by the COVID-19 pandemic, has forever shaped the future of GP training programmes, highlighting the need for a systematic, competency-based approach. The integration of telehealth and AI care has introduced new challenges of accessibility, efficiency, and clinical ingenuity, but also exposed gaps in competency development, ethical concerns, and dangers of depersonalized care (33–35). For the future of the UK, it is not a question of how to adjust to change, but how to propel the future of GP training so that it is both technologically outstanding and patient-centered.

There is one question at the heart of it all: do we train GPs to be improved clinicians, or just better efficient ones? Telehealth and AI definitely make it more efficient through effective workflows, reducing unnecessary clinic visit appointments, and expanded access to care. But the risk is that over-reliance on digital tools will diminish skills in clinical decision-making, reduce diagnostic confidence, and depersonalize the interaction with patients (36–39). The future of GP training has to resist this by keeping at the heart of primary care training face-to-face clinical judgment, human compassion, and professional instinct.

No less important is the role of lifelong learning in an AI-driven healthcare environment. The Hybrid GP Training Model cannot be seen as a one-off point of adjustment but as a constant process of upskilling and optimisation (6, 40, 41). AI, telehealth systems, and digital diagnosis will continue to evolve, and today's state-of-the-art will be obsolete tomorrow. GP trainees should be taught adaptive learning skills so that they learn to critically assess, absorb, and adapt to new technologies throughout their careers. “Digital fluency” should be as integral to GP training as clinical examination skills, enabling doctors to navigate new digital tools without forgetting the limitations of those tools.

The ethical dimensions of digital healthcare will also require ongoing scrutiny and fine tuning (31). If left unchecked, AI biases, digital disparities, and over-reliance on machine suggestions could widen disparities rather than narrow them. GP trainees should not just be trained as users of technology, but as ethical gatekeepers so that digital transformation is not gained at the cost of equity, privacy, or clinical integrity.

Finally, what kind of GP will we be training to be in the future? Future generations of GPs will need to be clinicians, digital navigators of healthcare, mental first-aiders, and public health leaders in a blended healthcare system (42, 43). They should be as skilled in AI-assisted diagnosis as in bedside medicine, as skilled at communication over the internet as at physical examination. Future GP training needs to be geared to this new reality so that technology enhances, rather than replaces, the art of medicine.

The pandemic presents an opportunity to redesign training in primary care, not just react to it (44, 45). With the integration of systematic telehealth training, AI-facilitated learning, interdisciplinary working, and ethical supervision within GP training, the UK is well-placed to set a global standard of medical education in the digital age. The challenge is not so much that telehealth and AI should be integrated into training, as how to use them to provide GPs who not just have technical skills, but also a deep respect for the human aspect of medicine.

We are at the threshold of medical education reform, and the future GP must be the bridge that ensures technology works in the service of medicine, not at its center—a vision supported throughout this viewpoint.
